# Neurodevelopmental outcome of preterm very low birth weight infants admitted to an Italian tertiary center over an 11-year period

**DOI:** 10.1038/s41598-021-95864-0

**Published:** 2021-08-11

**Authors:** Stefania Longo, Camilla Caporali, Camilla Pisoni, Alessandro Borghesi, Gianfranco Perotti, Giovanna Tritto, Ivana Olivieri, Roberta La Piana, Davide Tonduti, Alice Decio, Giada Ariaudo, Silvia Spairani, Cecilia Naboni, Barbara Gardella, Arsenio Spinillo, Federica Manzoni, Carmine Tinelli, Mauro Stronati, Simona Orcesi

**Affiliations:** 1 Neonatal Intensive Care Unit, Fondazione IRCCS Policlinico San Matteo, 27100 Pavia, Italy; 2 Child Neurology and Psychiatry Unit, Department of Brain and Behavioural Sciences, University of Pavia, 27100 Pavia, Italy; 3 Child Neurology and Psychiatry Unit, IRCCS Mondino Foundation, Via Mondino 2, 27100 Pavia, Italy; 4 Fondazione Stella Maris Mediterraneo, Chiaromonte, Potenza Italy; 5 IRCCS Fondazione Don Carlo Gnocchi, Milan, Italy; 6 Department of Neurology & Neurosurgery and Department of Diagnostic Radiology, Montreal Neurological Institute McGill University, Montreal, QC Canada; 7 Department of Obstetrics and Gynecology, IRCCS Foundation Policlinico San Matteo and University of Pavia, Pavia, Italy; 8 Department of Clinical, Surgical, Diagnostic and Paediatric Sciences, University of Pavia, Pavia, Italy; 9 Health Promotion - Environmental Epidemiology Unit, Hygiene and Health Prevention Department, Health Protection Agency, Pavia, Italy; 10 Clinical Epidemiology and Biometric Unit, IRCCS Policlinico San Matteo Foundation, Pavia, Italy

**Keywords:** Paediatric research, Neonatal brain damage, Neonatology, Paediatric research, Preterm birth

## Abstract

Preterm very low birth weight infants (VLBWi) are known to be at greater risk of adverse neurodevelopmental outcome. Identifying early factors associated with outcome is essential in order to refer patients for early intervention. Few studies have investigated neurodevelopmental outcome in Italian VLBWi. The aim of our longitudinal study is to describe neurodevelopmental outcome at 24 months of corrected age in an eleven-year cohort of 502 Italian preterm VLBWi and to identify associations with outcome. At 24 months, Griffiths’ Mental Developmental Scales were administered. Neurodevelopmental outcome was classified as: normal, minor sequelae (minor neurological signs, General Quotient between 76 and 87), major sequelae (cerebral palsy; General Quotient ≤ 75; severe sensory impairment). 75.3% showed a normal outcome, 13.9% minor sequelae and 10.8% major sequelae (3.8% cerebral palsy). Male gender, bronchopulmonary dysplasia, abnormal neonatal neurological assessment and severe brain ultrasound abnormalities were independently associated with poor outcome on multivariate ordered logistic regression. Rates of major sequelae are in line with international studies, as is the prevalence of developmental delay over cerebral palsy. Analysis of perinatal complications and the combination of close cUS monitoring and neurological assessment are still essential for early identification of infants with adverse outcome.

## Introduction

Preterm birth is a significant cause of infant and child mortality and morbidity. An estimated 11.1% of all births worldwide occur prematurely, with Europe recording the lowest rate (6.2%)^1^. In 2008 the annual birth number in Italy was of approximately 552,000 and preterm very low birth weight infants (VLBWi) account for 1% of this total^2^. In 2019 the annual birth number has decreased to 420.000 with the same percentage of VLBWi^3^.

Modern neonatal intensive care techniques have contributed to increased survival of infants born at the limit of viability^4,5^. A recent study of a large international cohort of very preterm infants born between 2007 and 2015 in 11 high-income countries showed that in the same period rates of mortality and of major comorbidities decreased in most of them, including Italy^6^.

Approximately 5–10% of all VLBWi present major motor deficits (cerebral palsy, CP), while 25–50% have cognitive, behavioral and/or attention disabilities. The impact of these deficits and disabilities can be huge, both on the individuals and families concerned, and on public health care resources, especially given the life-long nature of sequelae^7^.

The above considerations explain the growing importance of evaluating short- and long-term developmental outcome in premature infants admitted to neonatal intensive care units (NICUs).

To date, there are few Italian studies on neurodevelopmental outcome in large VLBWi cohorts: several recent papers have examined preterm infants’ neurodevelopment during early life, but the data are scarce, non-homogeneous, not comparable, and even seemingly contradictory. In a previous neurodevelopmental follow-up study in a sample of VLBWi born between 2005 and 2007 evaluated at 24 months of corrected age (CA) using Griffiths Mental Development Scales (GMDS-R), we found that 16% had neurological sequelae (11% minor and 5% major). Preterm premature rupture of the membranes, bronchopulmonary dysplasia (BPD), late-onset sepsis, postnatal steroid therapy and male gender were the most significant risk factors for adverse outcome^8,9^. Ionio et al.^10^ assessed cognitive and linguistic performance using the Bayley Scales of Infant and Toddler Development (BSID-III) in a population of healthy Italian preterm infants (mean gestational age 31.15 weeks) at 24 and 36 months of CA. They did not classify infants according to the neurodevelopmental outcome, but they highlighted significantly different scores between preterm and term infants at both 24 and 36 months. Moreover they found that lower gestational age, lower birthweight, and more days spent in hospital correlated with a larger gap between corrected and chronological scores at 24 months^10^. In a longitudinal study conducted in a large sample of monolingual preterm VLBWi without major cerebral damage, Sansavini et al.^11^ found that 20% showed delay in word production at 24 months of corrected age (CA), but cognitive profile was not investigated. They found that male gender, BPD and low maternal educational level increased the risk of language delay at 24 months^11^. The most recent Italian paper, published in 2020 by the Neuroprem working group, investigated neurodevelopmental outcome of 153 VLBWi at 2 years of age using GMDS-R or BSID III and neuro-functional evaluation according to the International Classification of Disability and Health (ICF-CY). In this regional prospective cohort study, the rate of severe functional disability was 8.5%, and the rate of CP was 4.5%. Associations with perinatal risk factors were not evaluated^12^.

A large body of follow-up data from a single Italian NICU (currently lacking) could constitute a valuable tool for assessing the quality of neonatal care provided, for establishing appropriate interventions and pursuing outcome improvements, and for making comparisons with other national and international NICUs.

The present longitudinal study aims to describe neurodevelopmental outcome at 24 months of corrected age (CA) in a large cohort of VLBWi admitted to a single Italian tertiary NICU, and to look for correlations between obstetric, perinatal and postnatal variables and short-term neurodevelopmental outcome.

## Materials and methods

A longitudinal study was performed at the NICU of the “Fondazione IRCCS Policlinico San Matteo” hospital in Pavia, Italy, in collaboration with the Department of Obstetrics and Gynecology of the same hospital and the Child Neurology and Psychiatry Unit at the IRCCS C. Mondino Foundation. All consecutive inborn and outborn VLBWi (weight ≤ 1500 g), admitted to the abovementioned NICU within 6 h of birth between January 1st 2005 and December 31st 2015, were eligible for inclusion in the present study. All were treated in accordance with state-of-the-art international, national and in-house guidelines^13^. All infants born at > 23 weeks GA received resuscitation in the delivery room and were subsequently transferred to the NICU. Infants born at extremely low gestational ages (< 23 weeks GA) showing strong respiratory effort and vital signs were transferred to the NICU after resuscitation, for subsequent palliative or full intensive care. Exclusion criteria were: major congenital malformations, diagnosis of a genetic disorder or congenital infection. The study was approved by the Ethical Committee Fondazione IRCCS Policlinico San Matteo. The parents of all the neonates signed the Institutional Informed Consent for the anonymous use of their children’s clinical data for scientific purposes.

### Data collection

Obstetric, perinatal and neonatal data were recorded and defined according to the Vermont Oxford Network database criteria for VLBWi^14^. For each newborn, the clinical risk index for babies (CRIB) score was calculated; the CRIB is a tool designed to quantify clinical severity on the basis of six variables evaluated during the first 12 h of life: birth weight (BW), gestational age (GA), minimum and maximum fraction of inspired oxygen to maintain a saturation of between 88 and 95%, worst base excess, presence of congenital abnormalities^15^.

### Ultrasound scan

In accordance with the protocol followed at our NICU, cerebral ultrasound (cUS), with serial examinations, was performed in all the preterm infants (from 2005 using an Acuson Sequoia 512; and from January 2014 using an Arietta V70)^16^. When detected, intraventricular hemorrhage (IVH) was classified according to Papile et al., periventricular leukomalacia (PVL) according to de Vries et al., and ventricular dilatation (VD) according to Ment et al.^17–19^. In accordance with the Rademaker classification, cUS scans performed at 40 weeks of post menstrual age (PMA) were classified as “normal” when they revealed minor or no abnormalities, “slightly abnormal” in the presence of grade I/II IVH, grade I PVL, germinal layer necrosis, or a combination of these features, or isolated VD, and “severely abnormal” in the presence of grade III/IV IVH, cystic grade II/III PVL, thalamic lesions, focal infarction, or post-hemorrhagic VD needing surgical intervention^20^.

### Neurological and neurodevelopmental assessment and follow up

A child neuropsychiatrist, unaware of the cUS findings, examined each subject at 38–42 weeks of PMA; neonatal neurological assessments were classified as “normal” or “abnormal” in the absence or presence of any pathological neurological sign, respectively^21^. Each infant underwent neurodevelopmental assessment every three months during the first year of life, and every 6 months in the second year^9,22^. The Griffiths Mental Development Scales (GMDS)^23^, were used to obtain each child’s General Quotient (GQ) at 24 months of CA. In addition, ophthalmological and audiometric examinations were performed periodically to exclude specific sensory abnormalities^9^.

At 24 months of CA, infants were classified into three categories according to their neurodevelopmental outcome: “normal” if they had a normal neurological assessment and a GQ ≥ 88; “minor sequelae” if they showed tone and reflex abnormalities or asymmetry without functional deficits, or at least one sign from the triad described by Amiel-Tison and Gosselin, GQ between 76 and 87, squint and refractive errors, mild hypoacusia; and “major sequelae” if they presented disabling or non-disabling CP, a GQ ≤ 75, sensorineural hearing loss requiring active intervention, or severe central or peripheral visual impairment^9^. CP diagnosis was performed by a trained neuropsychiatrist and defined and classified according to Rosenbaum et al. (2007)^24^.

### Statistical analysis

The continuous variables were reported as mean and standard deviation if normally distributed, and as median and interquartile range (IQR: 25th–75th percentile) if not normally distributed. The categorical variables were expressed as counts and percentages. The associations between categorical variables were studied with the Pearson’s χ^2^ test or with the Fisher’s exact test. For continuous variables, comparisons between two groups were performed with the Student’s t test or the analogous non parametrical test of Mann–Whitney, while for comparisons among more than two groups the ANOVA test or the analogous non parametrical test of Kruskal–Wallis were used. In order to study the association between risk factors and severity of neurological sequelae (normal, minor, major), univariate ordered logistic regression models were performed. The risk factors showing a *p* value < 0.2 on univariate analysis and no collinearity were included as regressors in a multivariate ordered logistic model. Appropriate tests for checking the ordered logistic regression assumptions (proportional odds assumption/parallel regression assumption) were performed; post hoc tests for fitting of the model were used, and the predicted values were graphically represented. Results were reported as odds ratio (OR) with corresponding 95% confidence interval. The significance level was set at alpha 0.05 (statistical significance for *p* values < 0.05). The statistical analysis was performed using STATA statistical software, version 14 or later (Stata Corporation, College Station, Texas, USA).

## Results

### Study population

**Figure 1:**
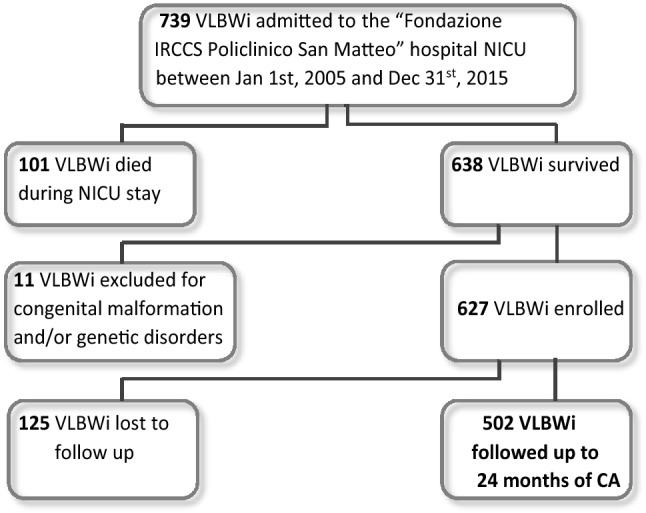
Participant flow diagram.

During the study period, 739 VLBWi were admitted to the NICU of the “Fondazione IRCCS Policlinico San Matteo” hospital and 728 eligible VLBWi, 647 inborn and 81 outborn infants, were included in the study. Of these, 101 (13.8%) died in the NICU. Both GA at birth and BW were significantly lower in the deceased group compared with the survivors with six out of eight infants with gestational age < 23 weeks dying in the first hours or within the first 10 days of life, and only two infants surviving to discharge (more details are shown in Supplementary Table 1). None of the infants died after discharge from the NICU. Eleven subjects (1.5%) were excluded from the present analysis on the basis of the exclusion criteria, while 125 infants (19.9%) were lost to follow up. No differences in perinatal medical data, cUS findings or neurological assessment at 40 weeks of PMA were found between the study group and the subjects lost to follow up (Supplementary Table 2). In total, 502 infants (80.1%) completed the follow-up evaluation and constitute the study group (Fig. 1).

The main sociodemographic, obstetric and neonatal characteristics of the study group are reported in Table 1, column 2. The cUS findings by neurological assessment at 40 weeks of PMA are shown in Supplementary Table 3.

**Table 1:** Clinical characteristics of the entire study sample and according to neurodevelopmental outcome.

	All infants	Normal outcome	Minor sequelae	Major sequelae	*p* value
	n = 502	n = 378	n = 70	n = 54	
**Maternal and obstetric characteristics**					
Maternal age, mean (SD), years^a^	32.7 (5.2)	32.5 (5.1)	33.87 (4.6)	32.57 (6.2)	0.272
Maternal education, mean (SD), years^a^					
≤ 5	8 (2.4)	8 (3.3)	0 (0)	0 (0)	
6–8	71 (22)	48 (20)	15 (33.3)	8 (21.6)	
9–13	164 (50.9)	124 (51.6)	22 (48.8)	18 (48.6)	
> 13	79 (24.5)	60 (25)	8 (17.7)	11 (29.7)	0.306
Assisted reproduction, n (%)	85 (16.9)	68 (17.9)	8 (11.4)	9 (16.7)	0.404
Multiple pregnancy, n (%)	139 (27.6)	109 (28.8)	15 (21.4)	15 (27.7)	0.445
Preeclampsia, n (%)^a^	94 (29.1)	77 (32)	10 (22.2)	8 (21.6)	0.074
PPROM > 48 h, n (%)^a^	38 (11.8)	26 (10.8)	7 (15.5)	5 (13.5)	0.733
Cesarean section, n (%)	411 (81.8)	312 (82.5)	53 (75.7)	46 (85.1)	0.316
Antenatal steroid therapy					
None	47 (9.36)	31 (8.2)	8 (11.43)	8 (14.81)	
Incomplete	380 (75.7)	293 (77.51)	50 (71.43)	37 (68.52)	
Complete	69 (13.75)	50 (13.23)	10 (14.29)	9 (16.67)	0.420
Histological chorioamnionitis^b^	111 (27.01)	74 (24.42)	22 (36.67)	15 (31.25)	0.443
Magnesium sulphate^a^	43 (9.84)	34 (10.33)	7 (11.66)	2 (4.17)	0.079
**Neonatal characteristics**					
Gestational age, mean (SD), weeks	29.12 (2.8)	29.41 (2.6)	28.6 (2.9)	27.81 (3.2)	< 0.001
Male, n (%)	246 (49)	176 (46)	37 (53)	35 (64.8)	0.028
Birth weight, mean (SD), g	1114.11 (290)	1140.64 (273.1)	1062.47 (303)	995.31 (349.6)	< 0.001
Small for gestational age, n (%)	90 (17.9)	60 (15.8)	19 (27.1)	11 (20.3)	0.044
CRIB, median (IQR)	1 (0–4)	1 (0–3)	1.5 (1–6)	2 (1–7)	0.001
Resuscitation, n (%)	377 (75.1)	269 (71.1)	57 (81.4)	51 (94.4)	< 0.001
Intubation, n (%)	252 (50.2)	173 (45.7)	40 (57.1)	39 (72.2)	< 0.001
Conventional ventilation, n (%)	256 (51)	179 (47.3)	37 (52.8)	40 (74.1)	0.001
Conventional ventilation, median (IQR), hours	48.5 (16–204)	48 (8.5–147)	49 (24–336)	84 (26.5–588)	0.004
HFO, n (%)	51 (10.1)	29 (7.6)	7 (10)	15 (27.7)	< 0.001
nCPAP, n (%)	415 (82.6)	301 (79.6)	63 (90)	51 (94.4)	0.006
nCPAP, median (IQR), hours	135 (48–540)	120 (48–408)	270 (72–720)	264 (75–600)	0.002
Surfactant, n (%)	251 (50)	177 (46.8)	37 (52.8)	37 (68.5)	0.010
Postnatal steroids, n (%)	55 (10.9)	29 (7.6)	15 (21.4)	11 (20.4)	< 0.001
Bronchopulmonary dysplasia, n (%)	117 (23.3)	68 (17.9)	24 (34.2)	25 (46.3)	< 0.001
Prophylaxis PDA, n (%)	95 (18.9)	63 (16.6)	15 (21.4)	17 (31.5)	0.028
Surgery PDA, n (%)	16(3.1)	9 (2.3)	2 (2.8)	5 (9.2)	0.026
NEC, n (%)	28 (5.5)	19 (5)	4 (5.7)	5 (9.2)	0.447
Early-onset sepsis, n (%)	3 (0.6)	3 (0.79)	0 (0)	0 (0)	0.609
Late-onset sepsis, n (%)	78 (15.5)	54 (14.2)	12 (17.1)	12 (22.2)	0.297
ROP grade ≥ 3, n (%)	31 (6.1)	15 (3.9)	6 (8.5)	10 (18.5)	< 0.001
Normal cUS findings according to Rademaker, n (%)	81 (16.1)	69 (18.2)	10 (14.2)	2 (3.7)	
Slightly abnormal cUS findings according to Rademaker, n (%)	378 (75.3)	294 (77.7)	53 (75.71)	31 (57.4)	
Severely abnormal cUS findings according to Rademaker, n (%)	43 (8.57)	15 (3.9)	7 (10)	21 (38.8)	< 0.001
Abnormal neurological examination at 40 weeks PMA, n (%)	162 (32.6)	98 (26.2)	32 (46.38)	32 (60.3)	< 0.001

*p* values for the comparison between the three groups are reported.

*PPROM* preterm prelabor rupture of membranes, *CRIB* clinical risk index for babies, *HFO* high-flow oxygen, *nCPAP* nasal continuous positive airway pressure, *PDA* Patent Ductus Arteriosus, *NEC* necrotizing enterocolitis, *ROP* retinopathy of prematurity, *cUS* cranial ultrasonography, *PMA* postmenstrual age.

^a^Data available only for the inborn subjects (n = 464).

^b^Data available only for the inborn subjects with placental histology (n = 411).

### Neurodevelopmental outcome

Neurodevelopmental outcome at 24 months of CA was classified as normal in 378 children (75.3%), while the remaining 124 (24.7%) presented neurological sequelae: classified as minor in 69 cases (13.9%) and major in 55 (10.8%) (Table 2). In the “major sequelae” group, 19 infants (3.8% of the whole sample) had CP: 9 of them had a non-disabling form and could walk unassisted, and 7 had a GQ > 75. Severe developmental delay was observed in 35 infants (7% of the whole sample).

**Table 2:** Neurodevelopmental outcome of the total study population (n = 502) at 24 months of CA.

Neurodevelopmental outcome	n (%)
**Normal**	378 (75.3)
**Minor sequelae**^**a**^	70 (13.9)
Stretch reflex	11 (2.2)
GQ between 76 and 87	42 (8.4)
Tone abnormalities	35 (7)
Stretch reflex and squint	1 (0.2)
Squamous suture	10 (2)
**Major sequelae**	54 (10.8)
Disabling CP	10 (2)
Non-disabling CP	9 (1.8)^b^
Severe visual impairment (peripheral or central)	4 (0.8)^c^
GQ ≤ 75 without other impairments	35 (7)

*GQ* general quotient, *CP* cerebral palsy.

^a^Each child can present more than one minor sequel.

^b^7 children had GQ > 75.

^c^3 children had CP and one had GQ < 75.

**Figure 2:**
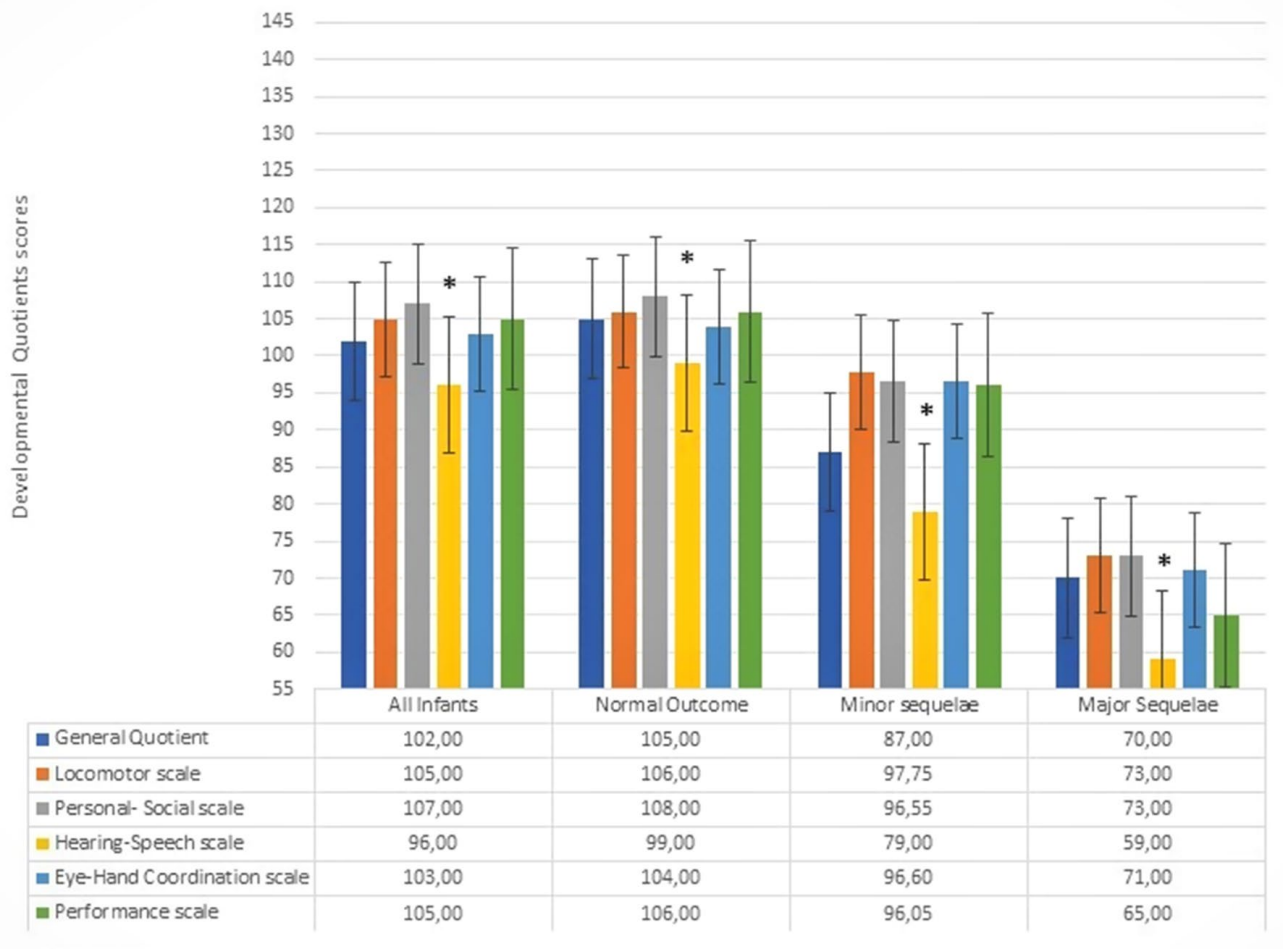
Developmental profile of the entire study sample and according to neurodevelopmental outcome categories at 24 months of CA. 95% CI. * = *p* < 0.001 among Hearing-Speech Scale and all other Scales in all groups of patients.

The GMDS findings are reported in Fig. 2. The median score on the Hearing-Speech subscale was significantly lower than the median scores recorded on all the other subscales (*p* < 0.001). This statistical significance persisted after restricting the analysis to the children with normal GQ (*p* < 0.001) (Fig. 2).

### Correlations between sociodemographic, obstetric and neonatal variables and outcome at 24 months of CA

Table 1 shows the sociodemographic, obstetric and neonatal variables divided by neurodevelopmental outcome and for each variable the corresponding p value for the statistical significance of the comparison between the three groups. Prenatal magnesium sulphate administration was introduced in routine prenatal care in 2008. We performed the univariate analysis, testing all the variables for association with neurodevelopmental outcome. Table 3 reports the results, in terms of OR and *p* value, of respectively the univariate and the multivariate analysis, with neurodevelopmental sequelae as outcome. The multivariate model was performed by including the variables for which no collinearity was reported. All the variables included in the multivariate model proved to be significantly associated with the outcome, even if, when adjusting for the other predictors included in the model, for each variable the significance level tended to be lower than in the univariate model. In the multivariate model, the highest OR was reported for severely abnormal cUS finding (OR 6.1; *p* value < 0.001); abnormal neurological assessment at 40 weeks of PMA showed a OR equal to 2.1 (*p* value 0.002). The presence of bronchopulmonary dysplasia reported an OR of 1.7 and the male gender an OR of 1.5, both statistically significant (respectively *p* = 0.026 and *p* = 0.043).

**Table 3:** Univariate and multivariate analysis of risk factors for adverse neurodevelopmental outcome.

	Univariate analysis			Multivariate analysis		
	OR	CI 95%	*p* value	OR	CI 95%	*p* value
Male	1.6	1.1–2.5	0.014	1.5	1.0–2.5	0.043
Bronchopulmonary dysplasia	3.0	1.9–4.7	< 0.001	1.7	1.1–2.9	0.026
Postnatal steroid treatment	2.9	1.6–5.0	< 0.001			
Clinical Risk Index for babies (CRIB)	1.1	1.0–1.2	< 0.001			
Retinopathy of prematurity	2.6	1.6–4.2	< 0.001			
Severely abnormal cUS findings (Rademaker classification)	14.1	6.0–33.0	< 0.001	6.1	2.4–15.1	< 0.001
Abnormal neurological examination at 40 weeks of PMA	3.2	2.1–5.0	< 0.001	2.1	1.3–3.4	0.002

*cUS* cranial ultrasonography, *PMA* postmenstrual age, *OR* odd ratio.

## Discussion

We have here reported the neurodevelopmental follow-up data recorded in a cohort of VLBWi consecutively admitted at birth to a tertiary Italian NICU over a recent 11-year period (2005 to 2015).

The single-center design of the study, despite its inherent limitations, ensured highly homogeneous standards of care and methodological consistency of data collection throughout. Both inborn and outborn patients were included in the study. Although poorly measurable, possible between-hospital differences in prenatal care and delivery room resuscitation protocols may have influenced the outcomes; however, outborn infants accounted for only 7.5% of the whole study population, and no statistically significant differences were found between inborn and outborn patients.

One hundred and one infants died in the NICU and were excluded from the analysis. They had a lower mean GA at birth and a lower mean BW compared with the survivors, suggesting a potentially greater burden of brain damage in the non survivors. In terms of mortality, higher at lower GAs^25^, and of major complications of prematurity, our study cohort is comparable to cohorts reported in national and international databases^14^.

The neurodevelopmental outcome analysis showed that 10.8% of the sample presented major neurological sequelae at 24 months of CA, with CP affecting 3.8%. Moreover, approximately half of the CP cases had a non-disabling form and could walk unassisted, and a considerable proportion of them showed satisfactory cognitive development. These findings, too, are in line with those of other authors^25^. The EPIPAGE study reported rates of survival without neurodevelopmental impairment at 2 years of corrected age of 48.5%, 90.0%, and 97.5% for infants born at 22–26, 27–31, and 32–34 weeks of gestational age respectively. The overall rate of CP at 24–26, 27–31, and 32–34 weeks' gestation was 6.9%, 4.3% and 1.0% respectively^26^. In a cohort of consecutive extremely preterm infants born before 27 weeks of gestation in Sweden between 2004 and 2007 and evaluated at 30 months of corrected age 42% children had no disability, 31% had mild disability, 16% had moderate disability, and 11% had severe disability. CP was present in 7%. Moderate or severe overall disability decreased with gestational age at birth^27^. The Victorian Infant Collaborative Study Group assessed neurodevelopmental outcome at 24 months of corrected age of infants born at gestational age 22–27 weeks in the state of Victoria in 1991 and 2005 and showed a rate of CP of 11% and 9.8% respectively^28^. The Epicure Study identified higher rates of CP (19%) at 30 months of corrected age, but they included preterm born ≤ 25 weeks gestation in the United Kingdom and Ireland in 1995^29^. This high percentage could be explained by the lower gestational age of the sample and the time of enrolment prior to 2000s. Differences in neurodevelopmental outcome rates, particularly regarding CP, could be related also on the different inclusion criteria between studies based on gestational age or on birthweight. When we started the recruitment, we chose birthweight according to VON criteria^14^. The mean gestational age of our cohort is of 29 weeks with only 10.5% SGA infants born > 32 weeks, nevertheless the presence of gestational age > 32 weeks could have influenced the outcome.

Consistent with the general trend shown by preterm infants born in the 2000s^7^, the major sequelae of preterm birth most frequently consisted of global developmental delay, rather than CP.

The impaired GQ scores recorded in our sample undoubtedly confirm the fragility of the cognitive development process in this population and the presence of emotional dysregulation^30^.

Our patients’ data showed a peculiar disharmonic neurodevelopmental profile at 24 months of CA, characterized by statistically significant impairment in the language domain, evident even in subjects with normal GQ^9^. Children born preterm are known to be at increased risk of long-term language impairments^31^, which can appear from early ages^32^, and last until school age or adolescence^33^. Our results confirm the findings from several previous studies suggesting that language impairment, detectable as early as 24 months of CA, is independent of cognitive disability^34^. The adverse neurodevelopmental outcome and peculiar disharmonic profile detected in our sample may be explained by an interplay of risk factors. According to the literature, there are several factors suggested to contribute to adverse language outcomes: environmental (a long NICU stay with non-optimal infant-caregiver interactions and underexposure to meaningful sensory stimuli), social (low maternal education level), biological (male gender), medical (BPD) and structural (altered brain maturation, atypical functional organization, delayed neural language lateralization and structural changes)^35,36^.

In brief, whereas global developmental delay, as expressed by a GQ ≤ 75, emerged as the most frequent major sequela, CP was less frequent, and less severe, than reported in the past, and affected subjects often showed satisfactory cognitive development. The cognitive profile of our very preterm infants was also characterized by significantly impaired language performance, even in the group without sequelae.

A further aim of our study was to identify potential associations with neurodevelopmental outcome in infants born in 2005 and later. In the present Italian study cohort, multivariate analysis identified male gender, BPD, cUS abnormalities and neurological assessment at 40 weeks of PMA as factors associated with neurodevelopment at 24 months of CA, in line with the findings of previous studies conducted in other cohorts.

In our cohort, social demographics did not affect neurodevelopmental outcome. This finding could be explained by the data availability because accurate social demographics were collected only for inborn infants. Moreover, the literature suggests that the influence of social demographics particularly affects cognitive domain and, possibly, 24 months of CA is too early to detect specific cognitive deficits and related risk factors^37^.

Magnesium sulphate was introduced as a prenatal treatment only in 2008 and was thus administered to only a minor proportion of infants. Our data, although not reaching statistical significance probably due to the low number of treated patients, appear to be consistent with previous observations showing association between magnesium sulphate administration and improved neurodevelopmental outcomes^38,39^.

Sex differences in preterm infants are known to influence both mortality and morbidity^40,41^. In particular, systematic reviews have shown that male gender per se is a prognostic factor for poorer cognitive development and language skills in early infancy (the “male disadvantage”), but not for CP in cohorts of children born very preterm^42^. The exact reason for this male vulnerability is still unclear; some authors have considered sex hormones and differences in sex chromosome gene expression as possible factors^42^, and others have explored a possible role of sex differences in microglia number, morphology and function in the onset and modulation of inflammation and in responses to pre and perinatal insults^43^. Moreover, neurological outcome appears to be more sensitive to suboptimal nutrition in male than in female preterm infants^44^. Overall, compared with females, male infants born very preterm appear to show a less efficient coping mechanism against stress factors, with consequences on brain development.

BPD is associated with a worse neurodevelopmental outcome, mostly motor dysfunction and poorer cognitive, language and neurosensory function^43^. In previous studies, motor and neurosensory and cognitive impairment in infants with BPD has been linked to delayed structural brain maturation^45–47^, and white matter microstructural abnormalities in the corpus callosum, corticospinal tract and superior cerebellar peduncle^48^. In our study, postnatal steroids were not associated with adverse neurodevelopmental outcomes. Other studies identified postnatal steroids as independent predictors of long-term neurodevelopmental outcomes^49^, but differences in timing and dosage of postnatal steroid administration and years of patients’ enrolment may account for differences across studies.

The multivariate analysis approach has previously identified the presence of lesions on cUS as strongly associated to neurodevelopmental outcome^7^. cUS remains the standard bedside examination in the NICU, and the most widely used technique for routine imaging, to detect IVH, monitor its progression, and follow up white matter injury and PVL evolution, especially in high-risk groups such as VLBWi^50^. In spite of its limits in identifying certain types of brain damage, such as diffuse white matter abnormalities and micro-cerebellar hemorrhages, and several disadvantages of the technique, such as interobserver and machine and probe type variability^51^, cUS is crucial for defining the neurodevelopmental prognosis, and thus for identifying infants needing to be referred for specialized follow-up and early intervention programs^52,53^. The strong association of brain lesions on cUS with the neurodevelopmental outcome confirmed in our study is possibly explained also by the close monitoring of patients during the first 4 weeks of life in our NICU, when cUS is routinely performed, as well as by the subsequent serial cUS monitoring, which is continued, during hospitalization, until patients reach 36 weeks of PMA. As suggested in the literature^54^, further follow-up evaluations are performed at 1–2 months of CA, or later, in order to better define the brain lesions, especially PVL.

Our results further confirm^9^, the current validity of traditional neurological assessment at term-equivalent age in preterm infants as significantly associated to the neurodevelopmental outcome: if pathological it is suggestive of major sequelae in the long term. Nevertheless, there remains a group of VLBWi with clear cognitive impairment that is not identified early by abnormal neurological assessment and/or cUs (i.e., in our cohort, 61% of the children with major sequelae did not present a severely abnormal cUS and 39% had a normal neurological assessment at term-equivalent age). In the context of “encephalopathy of prematurity”, advanced neuroimaging has already been applied to investigate the effects of dysmaturational disturbances of brain development^55^, with the aim of identifying, early on, children at greater risk of developing cognitive impairment^56–58^. Further studies are needed to find specific pre-perinatal factors associated to cognitive impairment that might be readily available to clinicians.

Finally, we underline that while the most severe motor sequelae and developmental delays can be diagnosed at 24 months, it is necessary to wait at least until school age in order to detect impairments (cognitive, behavioral and emotional) that may appear later. In this regard, our long-term follow-up is ongoing and will allow us to better highlight the characteristics of these preterm infants’ neurodevelopment and their resulting quality of life.

## Strengths and limitations

Our study is one of the few investigating the short-term neurodevelopmental outcome of VLBWi born in the 2000s in Italy^12^.

Overall, 80% of all the enrolled participants completed the follow up, a cut-off used to define high-quality randomized trials^59^, and no statistically significant differences in pre-perinatal characteristics were observed between the study group and the children lost to follow up.

The single-center design of the study meant that highly homogeneous standards of care were applied in all the enrolled participants throughout the study period and ensured methodological consistency of data collection during the follow up. On the other hand, maintaining the same methodology for a follow-up period of 10 years made our protocol less sensitive in identifying problems that are nowadays detected more frequently (for example autism spectrum disorders).

We are aware that exploration of general movements (GMs), if included in the neurological assessment performed at term-equivalent age, might have increased its prognostic value^60,61^. All the examiners involved in the present study are trained in GMs assessment, but due to objective difficulties in systematically obtaining video recordings and evaluating GMs in routine clinical practice, data on GMs trajectories are available only for a subgroup of our patients.

Moreover, the study design would be improved with a term-control reference group to compare some perinatal characteristics as well as term neurological assessments and 2-years outcome.

## Conclusion

Our study showed that, in a population of VLBWi born in Italy in the period 2005–2015, male gender, BPD, cUS monitoring and neurological examination at 40 weeks of PMA are strongly associated with the subsequent neurodevelopmental outcome. Furthermore, a combination of close cUS monitoring and neurological examination, in addition to evaluation of perinatal variables, proved essential for identifying infants needing to be referred for specific early intervention programs. Our data on neurodevelopmental outcome at 24 months of CA showed a rate of neurological impairment comparable to that reported in recent epidemiological literature from other countries.

## Supplementary Information

Supplementary Information.

## Data Availability

The datasets generated during and/or analyzed during the current study are available from the corresponding author on reasonable request.
