# Supplementary material for: Neurodevelopmental outcome of preterm very low birth weight infants admitted to an Italian tertiary center over an 11-year period
Source: Sci Rep. 2021 Aug 11;11:16316. doi: 10.1038/s41598-021-95864-0 (PMC8357917; doi:10.1038/s41598-021-95864-0)
Supplement: Supplementary file 1 — Supplementary Information. [file 41598_2021_95864_MOESM1_ESM.docx]

**Neurodevelopmental outcome of preterm very low birth weight infants admitted to an Italian tertiary center over an eleven-year period**

Stefania Longo ^1^, MD; Camilla Caporali* ^2,3^, MD; Camilla Pisoni ^1^, PsyD; Alessandro Borghesi ^1^, MD, PhD; Gianfranco Perotti ^1^, MD;  Giovanna Tritto ^2,4^, MD; Ivana Olivieri ^2, 5^, MD, PhD;  Roberta La Piana ^2, 6^, MD, PhD; Davide Tonduti ^2^, MD, PhD; Alice Decio ^2^, MD; Giada Ariaudo ^2, 3^, MD, PhD; Silvia Spairani ^2, 3^, MD; Cecilia Naboni ^3^, BS; Barbara Gardella ^7,8^, MD; Arsenio Spinillo ^7,8^, MD; Federica Manzoni ^9, 10^, MD; Carmine Tinelli ^9^, MD; Mauro Stronati ^1^, MD; Simona Orcesi ^2,3^, MD.

^1^ Neonatal Intensive Care Unit, Fondazione IRCCS Policlinico San Matteo, 27100 Pavia, Italy.

^2^ Child Neurology and Psychiatry Unit, Department of Brain and Behavioural Sciences, University of Pavia, 27100, Pavia, Italy.

^3^ Child Neurology and Psychiatry Unit, IRCCS Mondino Foundation, 27100 Pavia, Italy.

^4^ Fondazione Stella Maris Mediterraneo, Chiaromonte, Potenza, Italy

^5^ IRCCS Fondazione Don Carlo Gnocchi, Milan, Italy

^6^ Department of Neurology & Neurosurgery and Department of Diagnostic Radiology Montreal Neurological Institute McGill University, Montreal, QC, Canada

^7^ Department of Obstetrics and Gynecology, IRCCS Foundation Policlinico San Matteo and University of Pavia, Pavia, Italy.

^8^ Department of Clinical, Surgical, Diagnostic and Paediatric Sciences, University of Pavia, Pavia, Italy.

^9^ Clinical Epidemiology and Biometric Unit, IRCCS Policlinico San Matteo Foundation, Pavia, Italy.

^10^ Health Promotion - Environmental Epidemiology Unit, Hygiene and Health Prevention Department, Health Protection Agency, Pavia, Italy

**Corresponding author**: Camilla Caporali, MD, Child Neurology and Psychiatry Unit, IRCCS Mondino Foundation, via Mondino 2, 27100, Pavia, Italy. Phone number: +39-0382-380-287. E-mail address: camilla.caporali@mondino.it

**Supplementary table 1:** Clinical characteristics of the subjects died in NICU compared to survivors.

|  | **All infants** | **Survivors** | **Deaths in NICU** |
| --- | --- | --- | --- |
|  | **n= 739** | **n=638** | **n=101** |
| **Maternal and obstetric characteristics** |  |  |  |
| Assisted reproduction, n (%) | 108(14.84) | 96(15.31) | 12(11.88) |
| Multiple pregnancy, n (%) | 190(26.1) | 162(25.84) | 28(27.72) |
| Preeclampsia, n (%) ^a^ | 118(29.35) | 110(30.14) | 8(21.62) |
| **Neonatal characteristics** |  |  |  |
| Gestational age, mean (SD), weeks | 28.77(3.05) | 29.22(2.8) | 26.02(3.07) * |
| Male, n (%) | 373(51.24) | 316(50.4) | 57/56.44) |
| Birth weight, mean (SD), g | 1078,66(308.16) | 1127.15(286.11) | 777.64(267.78) * |
| Intubation, n (%) | 307(42.17) | 221(35.25) | 86(85.15) |
| Conventional ventilation, n (%) | 401(55.08) | 313(49.92) | 88(87.13) |
| HFO, n (%) | 103(14.15) | 59(9.41) | 44(43.56) * |
| nCPAP, n (%) | 532(73.08) | 517(82.46) | 15(14.85) |
| Surfactant, n (%) | 381(52.34) | 306(48.8) | 75(74.26) * |
| Postnatal steroids, n (%) | 72(9.89) | 65(10.37) | 7(6.93) |
| Bronchopulmonary dysplasia, n (%) | 146(20.05) | 139(22.17) | 7(6.93) * |
| Prophylaxis PDA, n (%) | 129(17.72) | 116(18.5) | 13(12.87) |
| Surgery PDA, n (%) | 19(2.61) | 17(2.71) | 2(1.98) |
| NEC, n (%) | 39(5.36) | 32(5.1) | 7(6.93) |
| Early-onset sepsis, n (%) | 14(1.92) | 3(0.48) | 11(10.89) * |
| Late-onset sepsis, n (%) | 44(6.04) | 37(5.9) | 7(6.93) |
| ROP grade ≥ 3, n (%) | 36(4.95) | 35(5.59) | 1 (0.99) * |

Abbreviations: HFO high-flow oxygen; nCPAP, nasal continuous positive airway pressure; PDA, Patent Ductus Arteriosus; NEC, necrotizing enterocolitis; ROP, retinopathy of prematurity.

* p value < 0.01

^a^ data available only for the inborn subjects (n = 464)

**Supplementary table 2:** Clinical characteristics of the subjects lost to follow-up compared to study group.

|  | **All infants** | **Study group** | **Drop out** |
| --- | --- | --- | --- |
|  | **n= 638** | **n = 502** | **n=125** |
| **Maternal and obstetric characteristics** |  |  |  |
| Assisted reproduction, n (%) | 96(15.31) | 85 (16.9) | 11(9.02) |
| Multiple pregnancy, n (%) | 162(25.84) | 139 (27.6) | 23(18.85) |
| Preeclampsia, n (%) ^a^ | 110(30.14) | 94 (29.1) | 16(37.20) |
| **Neonatal characteristics** |  |  |  |
| Gestational age, mean (SD), weeks | 29.22(2.8) | 29.12 (2.8) | 29.62(2.83) |
| Male, n (%) | 316(50.4) | 246 (49) | 55(45.08) |
| Birth weight, mean (SD), g | 1127.15(286.11) | 1114.11 (290) | 1181.12(265.87) |
| Intubation, n (%) | 221(35.25) | 252 (50.2) | 51(41.8) |
| Conventional ventilation, n (%) | 313(49.92) | 256 (51) | 54(44.26) |
| HFO, n (%) | 59(9.41) | 51 (10.1) | 8(6.56) |
| nCPAP, n (%) | 517(82.46) | 415(82.6) | 100(81.97) |
| Surfactant, n (%) | 306(48.8) | 251 (50) | 53(43.44) |
| Postnatal steroids, n (%) | 65(10.37) | 55 (10.9) | 10(8.2) |
| Bronchopulmonary dysplasia, n (%) | 139(22.17) | 117 (23.3) | 22(18.03) |
| Prophylaxis PDA, n (%) | 116(18.5) | 95 (18.9) | 21(17.21) |
| Surgery PDA, n (%) | 17(2.71) | 16(3.1) | 1(0.82) |
| NEC, n (%) | 32(5.1) | 28(5.5) | 4(3.28) |
| Early-onset sepsis, n (%) | 3(0.48) | 3 (.6) | 0(0) |
| Late-onset sepsis, n (%) | 37(5.9) | 78 (15.5) | 16(13.11) |
| ROP grade ≥ 3, n (%) | 35(5.59) | 31 (6.1) | 4(3.28) |
| Normal cUS findings according to Rademaker, n (%) | 124(20.06) | 81 (16.1) | 43(37.07) |
| Slightly abnormal cUS findings according to Rademaker, n (%) | 447(35.59) | 378 (75.3) | 69(59.48) |
| Severely abnormal cUS findings according to Rademaker, n (%) | 47(7.61) | 43 (8.57) | 4(3.45) |
| Abnormal neurological examination at 40 weeks PMA, n (%) | 189(35.59) | 162 (32.6) | 27(38.57) |

Abbreviations: HFO high-flow oxygen; nCPAP, nasal continuous positive airway pressure; PDA, Patent Ductus Arteriosus; NEC, necrotizing enterocolitis; ROP, retinopathy of prematurity; cUS, Cranial ultrasonography; PMA, postmenstrual age.

^a^ data available only for the inborn subjects (n = 464)

**Supplementary table 3**: Cerebral ultrasound ﬁndings and results of neurological assessment at 40 weeks of post menstrual age in the total study population (502 subjects)

|  | **cUS findings** | **Neurological assessment at 40 weeks of PMA ^a^** | |
| --- | --- | --- | --- |
|  | **n (%)** | **Normal** | **Pathological** |
| **Normal cUS** | 81 (16.1) | 63 (79.7) | 16 (20.3) |
|  |  |  |  |
| **Slightly abnormal cUS** | 378 (75.3) | 239 (67.5) | 115 (32.5) |
| Grade I PVL | 367 (73.1) |  |  |
| IVH I | 46 (9.1) |  |  |
| IVH II | 17 (3.3) |  |  |
| VD | 45 (8.9) |  |  |
| **Severely abnormal cUS** | 43 (8.5) | 12 (27.9) | 31 (72.1) |
| Grade II PVL | 19 (3.7) |  |  |
| Grade III PVL | 10 (1.9) |  |  |
| Grade IV PVL | 6 (1.2) |  |  |
| IVH III | 19 (3.7) |  |  |
| IVH IV | 4 (0.8) |  |  |
|  |  |  |  |
| **Abbreviations: cUS,** cranial ultrasonography; PVL, periventricular leukomalacia; IVH, intraventricular hemorrhage; VD, ventricular dilatation  **^a^** data unavailable for 26 subjects (5.17%) | | | |
